# Plaque Vulnerability as Assessed by Radiofrequency Intravascular Ultrasound in Patients with Valvular Calcification

**DOI:** 10.1371/journal.pone.0165885

**Published:** 2016-11-02

**Authors:** Nagendra Boopathy Senguttuvan, Sharath Kumar, Wang-Soo Lee, Sundeep Mishra, Jun Hwan Cho, Jee Eun Kwon, Seong Hyeop Hyeon, Yun Sang Jeong, Hoyoun Won, Seung Yong Shin, Kwang Je Lee, Tae Ho Kim, Chee Jeong Kim, Sang-Wook Kim

**Affiliations:** 1 Heart Research Institute, Chung-Ang University Hospital, Seoul, 06973, Korea; 2 All India Institute of Medical Sciences, New Delhi, 110029, India; Shenzhen Institutes of Advanced Technology, CHINA

## Abstract

**Background:**

Cardiac valvular calcification is associated with the overall coronary plaque burden and considered an independent cardiovascular risk and prognostic factor. The purpose of this study was to evaluate the relationship between the presence of valvular calcification and plaque morphology and/or vulnerability.

**Methods:**

Transthoracic echocardiography was used to assess valvular calcification in 280 patients with coronary artery disease who underwent radiofrequency intravascular ultrasound (Virtual Histology IVUS, VH-IVUS). A propensity score–matched cohort of 192 patients (n = 96 in each group) was analyzed. Thin-capped fibroatheroma (TCFA) was defined as a necrotic core (NC) >10% of the plaque area with a plaque burden >40% and NC in contact with the lumen for ≥3 image slices. A remodeling index (lesion/reference vessel area) >1.05 was considered to be positive.

**Results:**

Patients were divided into two groups: any calcification in at least one valve (152 patients) vs. no detectable valvular calcification (128 patients). Groups were similar in terms of age, risk factors, clinical diagnosis, and angiographic analysis after propensity score-matched analysis. Gray-scale IVUS analysis showed that the vessel size, plaque burden, minimal lumen area, and remodeling index were similar. By VH-IVUS, % NC and % dense calcium (DC) were greater in patients with valvular calcification (p = 0.024, and p = 0.016, respectively). However, only % DC was higher at the maximal NC site by propensity score-matched analysis (p = 0.029). The frequency of VH-TCFA occurrence was higher depending on the complexity (p = 0.0064) and severity (p = 0.013) of valvular calcification.

**Conclusions:**

There is a significant relationship between valvular calcifications and VH-IVUS assessment of TCFAs. Valvular calcification indicates a greater atherosclerosis disease complexity (increased calcification of the coronary plaque) and vulnerable coronary plaques (higher incidence of VH-TCFA).

## Introduction

Heart valve calcification is often observed upon ultrasound examination of the heart as the average life span of patients has increased. The presence of valvular calcifications is considered a manifestation of inflammation and diffuse atherosclerosis. In particular, mitral annular calcification (MAC) and aortic valve calcification (AVC) are associated with independent cardiovascular risk factors; such as coronary artery calcification, incidence of cardiovascular disease events, and both cardiovascular and all-cause mortality [1–6].

Valvular calcification has been associated with coronary plaque burden when observed using coronary angiography or non-contrast computed tomography [7,8]. However, the impact of multiple heart valve calcium deposits on plaque components and the vulnerability of the coronary arteries are not yet known. Assessment using virtual histology intravascular ultrasound (VH-IVUS) is widely performed and is a very useful tool for observing plaque characteristics in current clinical practice settings.

Thus, the objective of this study was to evaluate the relationship between the presence of valvular calcification and the plaque morphology and/or vulnerability of target lesions using transthoracic echocardiography (TTE) and VH-IVUS in patients with coronary artery disease (CAD).

## Methods

### Study population

This was an exploratory study utilizing retrospective analysis. The study protocol was approved by the Ethics Committee and Institutional Review Board at Chung-Ang University Hospital and was conducted in accordance with the Declaration of Helsinki. Owing to the retrospective nature of the study, the need for verbal or written patient consent was waived. However, patient records and informations were anonymized and deidentified prior to analysis.

We retrospectively assessed the records of 280 patients with suspected or known CAD who underwent both TTE and coronary angiography with VH-IVUS within a one month period (S1 Table). Finally, a propensity score-matched cohort of 192 patients (n = 96 in each group) was chosen as the primary data set. Standard coronary risk factors were collected including age, sex, hypertension (systolic blood pressure ≥140 mmHg or diastolic blood pressure ≥90 mmHg, or current use of antihypertensive medication), diabetes mellitus (treatment for diabetes mellitus, a fasting serum glucose ≥ 126 mg/dL, or hemoglobin A_1_C ≥ 6.5mg/dL), hypercholesterolemia (total cholesterol level ≥240 mg/dL or treatment for hypercholesterolemia), current smoking status (within the past 12 months), and family history of CAD (myocardial infarction in a first-degree relative aged <60 years).

### Angiographic analysis

Coronary angiography was performed after an intracoronary injection of 200 μg nitroglycerin. All angiograms were analyzed using an automated edge-detection algorithm (AI-1000 century, GE) and standard protocols. Culprit lesion length, reference vessel diameter, minimal lumen diameter, percent diameter stenosis, and the Thrombolysis in Myocardial Infarction (TIMI) grade were measured from end-diastolic frames. Target lesions for angiography were identified according to the clinical presentation of the patient and electrocardiographic, echocardiographic, or myocardial perfusion on angiographic findings.

### Echocardiographic analysis

**Fig 1:**
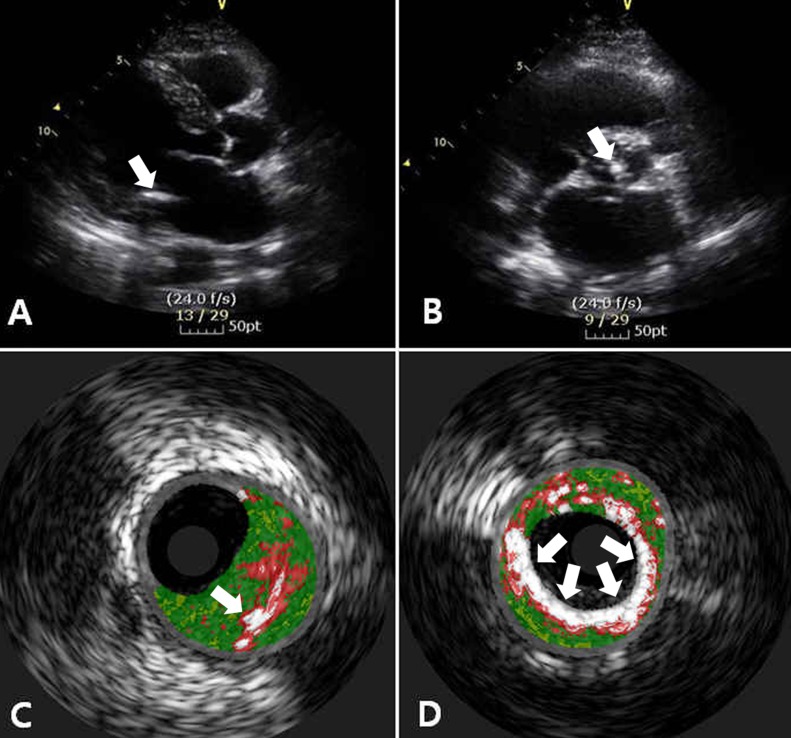
Representative images of valvular calcification and coronary artery calcification(white arrows). Transthoracic echocardiography showed mitral annular calcification (A) and aortic valve calcification (B). VH-IVUS demonstrated mild and severe coronary calcification. Mild valvular calcification revealed the prominent fibrous (green color) plaque components (C), while severe valvular calcification showed densely calcified plaque (D).

A complete 2-dimensional (2-D) echocardiogram at rest was obtained for all patients using an iE33 Xmatrix (Philips, Andover, MA, USA) with a 3.5-MHz transducer. All standard views were obtained during quiet respiration. Three experienced sonographers conducted the sonographic measurements while two cardiologists (NBS, JEK) who were blinded to the clinical and laboratory information evaluated the results. MAC was defined as a highly reflective area with acoustic shadowing located at the junction of the atrioventricular groove and the posterior mitral leaflet on the parasternal long-axis, apical 4-chamber, or parasternal short-axis views (Fig 1A) [9].

AVC was defined as bright, dense echoes greater than or equal to 1 mm in size on one or more cusps (Fig 1B) (2). The severity of aortic or mitral valve leaflet and annular calcification was graded as ‘mild to moderate’ when deposits were thin (1~4 mm) and as ‘severe’ when deposits were thick (>4mm). The thickness of the anterior and posterior mitral valve leaflets were measured as the mean of three consecutive 2-D measurements, as previously described [10].

### Grayscale and virtual histology intravascular ultrasound imaging

A commercially available VH-IVUS system (Volcano Therapeutics, Rancho Cordova, CA) and 20 MHz transducer were used for all IVUS examinations. The IVUS catheter was advanced 10 mm distal to the target lesion and imaging was performed back to the aorto-ostial junction using an ECG-gated automatic pullback device. Studies were recorded on electronic media for offline analysis at a later stage. Gray-scale IVUS analysis was performed according to the criteria laid out by the American College of Cardiology clinical expert consensus document on IVUS and included the external elastic membrane, the lumen, plaque and media (external elastic membrane minus lumen) areas [11]. VH-IVUS imaging was performed prior to any intervention and after intracoronary administration of 200 μg nitroglycerin. VH-IVUS analysis classified the intracoronary plaques as fibrotic tissue (FT), fibrofatty (FF), necrotic core (NC), and dense calcium (DC). Plaque compositions were expressed with colors (green for FT, yellow for FF, red for NC, and white for DC). Measurements were performed over the entire length. Virtual histology thin-capped fibroatheroma (VH-TCFA) was defined as NC >10% of the plaque area with a plaque burden >40% and in contact with the lumen for ≥3 image slices [12]. The remodeling index was defined as the ratio of external elastic membrane cross-sectional area at the target lesion to that at the proximal reference. Positive remodeling was defined as a remodeling index >1.05 [13].

### Statistical analysis

Continuous data were presented as the mean ± SD or median (interquartile range). Categorical variables were presented as numbers and percentages. Groups were compared for categorical data or frequency of events using Fisher’s exact test or the χ^2^ test; and for continuous variables using an unpaired Student's t-test, the Kruskal-Wallis test, and ANOVA. Post hoc testing was performed using Tukey’s test or the Steel-Dwass method for variables with and without normal distribution. Propensity score-matched analysis was used to adjust for differences between the groups. Age, gender, risk factors, clinical diagnosis, and angiographic findings were adjusted for the propensity score-matched model. After propensity score-matched analysis, we analyzed the baseline covariates between the groups. All tests were two-sided, and a P-value <0.05 was considered statistically significant. All statistical analysis was performed using SPSS (Version 20.0, SPSS Inc., Chicago, IL).

## Results

### Clinical and angiographic findings

Clinical patient characteristics are presented in Table 1. Of 280 patients, valvular calcification was observed in 152 patients, while 128 patients had no detectable valvular calcification. The mean age of patients in any calcification group was higher than that of patients without valvular calcification (p<0.001). Any calcification group included a greater number of diabetic (p = 0.014) and hypertensive (p = 0.030) patients than those with no detectable valvular calcification. After propensity score-matched analysis, clinical characteristics between groups did not differ. Smoking status, hyperlipidemia, creatinine, and body mass indices were similar in both groups. Both clinical presentation and extent of CAD were also found to be similar. Quantitative angiographic analysis showed that the reference vessel diameter was larger in any calcification group than in those patients without calcification (p = 0.001). However; lesion length, minimal lumen diameter, and diameter stenosis showed no differences between the two groups. After propensity score-matched analysis, the reference vessel diameters were found to be of similar length (p = 0.47).

**Table 1:** Clinical and angiographic characteristics.

	Overall population			Propensity-matched population		
	Any calcification (N = 152)	No detectable calcification (N = 128)	p-value	Any calcification (N = 96)	No detectable calcification (n = 96)	p-value
Age (year)	62.72±11.19	57.75±10.77	<0.001	60.03±10.80	59.04±10.46	0.520
Sex (% of male/female)	72.4/27.6	84.4/15.6	0.016	82.3/17.7	80.2/19.8	0.712
Diabetes (%)	27.0	14.8	0.014	21.9	16.8	0.379
Hypertension (%)	48.0	35.2	0.030	44.8	41.7	0.662
Smoking (%)	42.8	45.3	0.669	54.2	40.6	0.060
Total cholesterol(mg/dl)	184.47±42.55	189.63±44.02	0.324	182.60±42.29	189.00±43.64	0.309
High density cholesterol (mg/dl)	43.68±10.65	43.78±9.96	0.936	42.72±9.57	44.33±10.34	0.268
Low density cholesterol (mg/dl)	114.08±35.56	115.75±36.10	0.700	112.90±35.42	115.30±35.61	0.635
Triglycerides (mg/dl)	125.63±73.49	156.99±116.37	0.010	128.90±71.05	159.60±128.70	0.054
Creatinine (mg/dl)	1.11±0.87	1.00±0.26	0.142	0.98±0.24	1.01±0.27	0.376
BMI (kg/m^2^)	23.98±2.90	24.12±2.46	0.668	24.04±2.59	24.05±2.26	0.994
Diagnosed coronary artery disease, n			0.796			0.502
stable angina	19	20		9	14	
unstable angina	30	25		21	18	
myocardial infarction	103	83		65	63	
NO. of diseased coronary arteries, n			0.228			0.814
1 /2 /3	0/64/44/44	1/64/38/25		35/31/25	40/30/23	
Pre-intervention						
Lesion length (mm)	19.79±9.65	20.12±9.43	0.797	20.12±9.59	20.98±9.56	0.542
Minimal lumen diameter (mm)	0.66±0.58	0.80±0.70	0.133	0.81±0.62	0.78±0.67	0.709
Reference vessel diameter(mm)	4.48±2.91	3.52±1.41	0.001	3.14±0.64	3.23±0.86	0.465
Diameter stenosis (%)	76.97±20.73	75.82±19.93	0.691	73.07±20.55	76.00±19.43	0.322

Values are presented as means±SD or n(%). BMI; body mass index.

### Echocardiographic and intravascular ultrasound measurements

The study population was divided into groups according to the presence and severity of calcification in the aortic or mitral valve leaflets and MAC. At least one valvular calcification was observed in 152 patients, while 128 patients had no detectable valvular calcification. Of the 152 patients with any calcification, both AVC and MAC were observed in 52 patients, while isolated calcifications were observed in 100 patients (MAC alone, 31 patients; AVC alone, 69 patients). Calcification was classified as mild to moderate in 76 patients, while the remainder of the group was classified as severe.

Gray-scale IVUS measurements are presented in Table 2. The vessel size, lesion length, and the minimal lumen area were similar in both groups. The average plaque burden was larger in any valvular calcification group than in those without calcification (p = 0.036), however, the size was found to be similar after propensity score-matched analysis (p = 0.6930). Although the remodeling index of minimal lumen area site was similar, positive remodeling was prominent at the maximal NC site in both groups (p = 0.88). Positive remodeling was observed in 61.2% (93/152) of patients in any calcification group and 57% (73/128) of patients in those without calcification (p = 0.38).

**Table 2:** IVUS measurements according to the presence of valvular calcification.

	Overall population			Propensity-matched population		
	Any calcification (n = 152)	No detectable calcification (n = 128)	p-value	Any calcification (n = 96)	No detectable calcification (n = 96)	p-value
Proximal reference						
EEM area (mm^2^)	17.15±5.35	17.10±5.71	0.944	16.96±5.87	17.37±5.46	0.631
lumen area (mm^2^)	8.29±2.58	8.93±3.52	0.093	8.47±2.64	8.86±3.59	0.403
plaque area (mm^2^)	8.86±3.91	8.17±3.62	0.136	8.49±4.13	8.51±3.32	0.970
Lesion site						
lesion length (mm)	19.58±8.22	19.53±9.27	0.964	19.61±8.62	20.79±9.90	0.379
EEM area (mm^2^)	14.34±5.10	14.30±5.53	0.949	14.10±5.48	14.46±5.70	0.654
minimal lumen area (mm^2^)	2.98±1.51	3.18±1.90	0.336	3.07±1.64	3.20±2.03	0.650
plaque area (mm^2^)	9.18±3.79	8.85±3.77	0.470	8.85±4.13	9.04±3.81	0.740
plaque burden (%)	62.81±8.88	60.57±8.90	0.036	60.95±8.99	61.46±8.98	0.693
Maximal necrotic core site						
EEM area (mm^2^)	16.56±5.78	15.96±5.60	0.384	16.19±6.26	16.21±5.55	0.980
lumen area (mm^2^)	4.71±2.33	4.88±2.91	0.586	4.91±2.51	5.05±3.18	0.738
plaque area (mm^2^)	11.85±4.78	11.24±4.63	0.283	11.28±5.01	11.37±4.28	0.895
plaque burden (%)	71.06±10.70	69.69±11.72	0.311	69.27±10.38	69.93±11.18	0.671
remodeling index	1.13±0.34	1.13±0.29	0.881	1.19±0.38	1.16±0.35	0.595
Distal reference segment						
EEM area (mm^2^)	12.38±5.30	12.44±5.39	0.924	12.29±5.53	12.39±5.46	0.894
lumen area (mm^2^)	6.93±2.86	7.21±3.12	0.448	7.06±2.95	6.99±3.05	0.879
plaque area (mm^2^)	5.45±3.12	5.21±3.05	0.537	5.23±3.27	5.37±3.19	0.759
**VH-IVUS measurements**						
**Lesion segment**						
mean fibrotic plaque (mm^2^)	3.72±1.88	3.89±2.36	0.494	3.58±2.06	3.93±2.39	0.272
mean fibrofatty plaque (mm^2^)					0.69±0.54	0.71±0.70	0.818	0.64±0.53	0.76±0.77	0.212
mean necrotic core (mm^2^)	1.52±1.14	1.26±0.84	0.031	1.41±1.16	1.29±0.85	0.423
mean dense calcium (mm^2^)	0.60±0.58	0.43±0.34	0.002	0.53±0.55	0.44±0.34	0.199
mean fibrotic plaque (%)	58.68±9.86	60.90±9.99	0.063	59.18±9.12	60.55±9.56	0.308
mean fibrofatty plaque(%)	10.28±5.37	10.83±6.44	0.439	10.11±5.22	11.33±7.07	0.178
mean necrotic core (%)	21.36±8.25	19.09±8.43	0.024	20.74±7.98	19.36±8.62	0.250
mean dense calcium (%)	8.70±5.80	7.07±5.40	0.016	8.31±5.54	7.37±5.71	0.251
**Maximal necrotic core site**						
fibrotic plaque (mm^2^)	4.29±2.32	4.61±2.69	0.279	4.06±2.37	4.56±2.32	0.146
fibrofatty plaque (mm^2^)	0.69±0.80	0.59±0.56	0.243	0.59±0.65	0.62±0.58	0.748
necrotic core (mm^2^)	3.06±1.87	2.63±1.73	0.048	2.94±1.99	2.71±1.80	0.406
dense calcium (mm^2^)	1.13±1.06	0.76±0.68	0.001	1.02±0.90	0.78±0.65	0.038
fibrotic plaque (%)	47.77±12.11	53.49±12.04	<0.0001	47.87±11.89	52.78±11.71	0.004
fibrofatty plaque (%)	6.76±6.05	6.86±5.71	0.880	6.34±5.52	7.09±6.11	0.379
necrotic core (%)	33.37±11.08	30.37±10.80	0.023	33.75±11.25	30.62±11.11	0.054
dense calcium (%)	12.12±8.50	9.26±8.13	0.004	12.05±7.75	9.51±8.25	0.029

### Plaque morphology, vulnerability, and valvular calcification

The mean % NC and % DC for any valvular calcification group were larger at both the lesion site (p = 0.024, p = 0.016, respectively) and the maximal NC site (p = 0.023, p = 0.004, respectively) than in those without valvular calcification. However, after propensity score-matched analysis, only % DC was larger in the valvular calcification group than in the group without calcification (p = 0.029). The fibrotic plaque was larger at the maximal NC site in the group without calcification (p<0.0001) (Table 2). With regards to the severity of calcification, % mean NC was higher (p = 0.049) and % mean DC was larger (p = 0.003) at the lesion segment in the severe calcification group. In addition, the NC and DC of the maximal NC site were also found to be larger in the severe calcification group (p = 0.042, p = 0.006, respectively). The % fibrotic plaque was higher in the group without calcification (p<0.0001) when compared to the group with calcification. In the calcification group, the NC and DC were similar between the group with isolated calcification and that with combined AVC and MAC. After propensity score-matched analysis, only the DC area (p = 0.008) and % DC (p = 0.047) were higher at the maximal NC site when comparing the three groups (Table 3, Fig 1C and 1D).

**Table 3:** VH-IVUS analysis depending on valvular calcification after propensity score matching analysis.

	**No detectable calcification (n = 96)**	**Isolated calcification (n = 63)**	**Combined presence of AVC and MAC (n = 33)**	3 Groupp-value	2 Groupp-value
**Lesion segment**					
mean fibrotic plaque (mm^2^)	3.93±2.39	3.70±2.26	3.35±1.62	0.253	0.389
mean fibrofatty plaque (mm^2^)	0.76±0.77	0.69±0.59	0.55±0.38	0.148	0.162
mean necrotic core (mm^2^)	1.29±0.85	1.54±1.33	1.17±0.71	0.259	0.080
mean dense calcium (mm^2^)	0.44±0.34	0.57±0.61	0.45±0.39	0.661	0.274
mean fibrotic plaque (%)	60.55±9.56	58.33±9.39	60.79±8.48	0.535	0.212
mean fibrofatty plaque (%)	11.33±7.07	10.10±5.11	10.14±5.49	0.561	0.967
mean necrotic core (%)	19.36±8.62	21.23±8.17	19.81±7.63	0.857	0.411
mean dense calcium (%)	7.37±5.71	8.47±5.55	7.99±5.59	0.867	0.687
**Maximal Necrotic core site**					
fibrotic plaque (mm^2^)	4.56±2.32	4.20±2.60	3.79±1.86	0.166	0.375
fibrofatty plaque (mm^2^)	0.62±0.58	0.64±0.71	0.49±0.52	0.245	0.282
necrotic core (mm^2^)	2.71±1.80	3.10±2.21	2.64±1.49	0.541	0.236
dense calcium (mm^2^)	0.78±0.65	1.06±0.97	0.95±0.78	0.717	0.568
fibrotic plaque (%)	52.78±11.71	47.51±11.09	48.56±13.44	0.356	0.682
fibrofatty plaque (%)	7.09±6.11	6.47±5.37	6.12±5.87	0.516	0.770
necrotic core (%)	30.62±11.11	33.99±11.07	33.30±11.73	0.534	0.779
dense calcium (%)	9.51±8.25	12.06±7.52	12.05±8.29	0.324	0.996
	**No detectable calcification (n = 96)**	**Mild to moderate Calcification (n = 48)**	**Severe calcification (n = 48)**	3 Group p-value	2 Group p-value
**Lesion segment**					
mean fibrotic plaque (mm^2^)	3.93±2.39	3.55±2.35	3.60±1.74	0.594	0.894
mean fibrofatty plaque (mm^2^)	0.76±0.77	0.69±0.62	0.59±0.42	0.188	0.363
mean necrotic core (mm^2^)	1.29±0.85	1.42±1.16	1.40±1.18	0.699	0.936
mean dense calcium (mm^2^)	0.44±0.34	0.44±0.33	0.61±0.69	0.029	0.119
mean fibrotic plaque (%)	60.55±9.56	57.82±8.92	60.53±9.20	0.571	0.147
mean fibrofatty plaque (%)	11.33±7.07	10.80±6.00	9.42±4.25	0.096	0.198
mean necrotic core (%)	19.36±8.62	21.18±8.19	20.30±7.82	0.808	0.593
mean dense calcium (%)	7.37±5.71	7.98±5.41	8.63±5.70	0.262	0.570
**Maximal Necrotic core site**					
fibrotic plaque (mm^2^)	4.56±2.32	4.19±2.65	3.94±2.07	0.208	0.611
fibrofatty plaque (mm^2^)	0.62±0.58	0.64±0.77	0.54±0.51	0.384	0.430
necrotic core (mm^2^)	2.71±1.80	2.95±1.97	2.94±2.04	0.647	0.980
dense calcium (mm^2^)	0.78±0.65	0.88±0.68	1.16±1.07	0.008	0.126
fibrotic plaque (%)	52.78±11.71	48.73±10.87	47.01±12.89	0.027	0.481
fibrofatty plaque (%)	7.09±6.11	6.28±5.30	6.41±5.78	0.674	0.911
necrotic core (%)	30.62±11.11	33.60±9.62	33.90±12.78	0.224	0.897
dense calcium (%)	9.51±8.25	11.40±7.39	12.71±8.11	0.047	0.413

3 Group p-value: no calcification vs. mild to moderate vs. severe calcification, ANOVA test. 2 Group p-value: mild to moderate vs. severe calcification, T-test.

**Fig 2:**
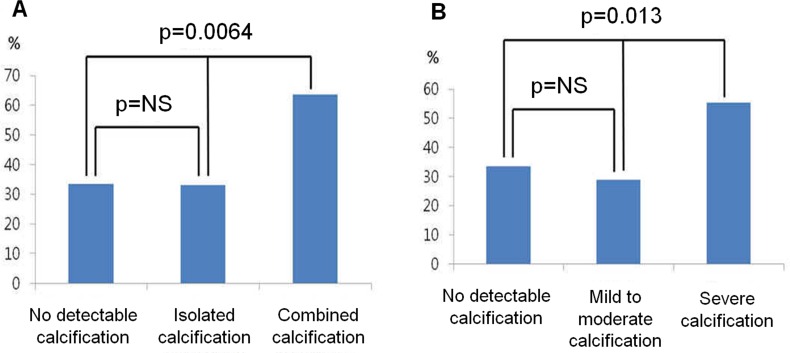
**Incidence of VH-TCFA according to the complexity (A) and severity (B) of valvular calcification.** A: Patients with combined aortic and mitral valve calcification had more VH-TCFAs than those with either no detectable valvular calcification or isolated calcification. B: Patients with severe valvular calcification had a higher incidence of VH-TCFAs than those with either no detectable valvular calcification or mild to moderate calcification.

Overall, VH-TCFA was identified in 38.6% (108/280) of the total study population. The incidence of VH-TCFA was higher depending on the complexity of calcification: 34.4% (44/128) in patients with no detectable calcification, 33% (33/100) in patients with isolated calcification, and 63.5% (31/52) in patients with combined AVC and MAC. Patients with combined AVC and MAC were found to have more VH-TCFAs than those with either no detectable valvular calcification or isolated calcification (p = 0.003). The association between a higher incidence of VH-TCFAs and the complexity of calcification was confirmed after propensity score-matched analysis (p = 0.0064) (Fig 2A, S2 Table).

According to the severity of valvular calcification, VH-TCFA occurred in 34.4% (44/128) of patients with no detectable calcification, 28.9% (22/76) of patients with mild to moderate calcification, and 55.3% (42/76) of patients with severe calcification. VH-TCFA was observed more frequently in patients with severe valvular calcification than in those with either no detectable valvular calcification or mild to moderate calcification (p = 0.002). After propensity score-matched analysis, the incidence of VH-TCFAs was higher in patients with severe valvular calcification (p = 0.0130) (Fig 2B, S2 Table).

## Discussion

There were three major findings in the present study: that valvular calcification was frequently combined (MAC and AVC) in acute coronary syndrome (ACS); that the presence of valvular calcification was an indicator of lesion complexity with more calcified coronary plaque and VH-TCFA, suggestive of lesion instability; and that both the complexity and severity of valvular calcification were associated with a high frequency of VH-TCFA occurrence.

The relationship between cardiovascular risk factors and CAD was first suggested in the Framingham study using an epidemiological approach [14,15]. Both invasive and non-invasive techniques are used to explore the correlation between cardiovascular risk factors and morphological characteristics of atherosclerotic plaques [16,17–19]. Two-dimensional echocardiography is the most commonly used imaging technique for the evaluation of cardiac function. Echocardiographic measurements provide powerful prognostic information for cardiovascular outcomes, such as the presence of left ventricular hypertrophy, aortic stenosis, and left ventricular ejection fraction [20,21]. Moreover, cardiovascular risk may be stratified using standard TTE, by means of the calcification score index, the Duke score, the Framingham risk score, and so forth [21–23]. While the prevalence of AVC and MAC does increase with age, it is also strongly related to traditional cardiovascular risk factors; including diabetes mellitus, hypertension, hyperlipidemia, and metabolic syndrome [6–8]. As an invasive modality, IVUS provides tomographic visualization and quantifies atherosclerotic plaque volume and burden. The major advantage of VH-IVUS is its practicality for use in a clinical setting and real-time assessment of plaque morphology. In the present study, valvular calcification was made up of both MAC and AVC in patients of an older age and those with diabetes mellitus and hypertension. Valvular calcification was frequently combined in ACS, and severe calcification of the cardiac valves was observed in half of these patients.

It has previously been shown that valvular calcification is not a merely a bystander to disease, but rather an indication of more vulnerable plaques [16]. Coronary calcification has been shown to be predictive of cardiovascular disease mortality; whereas calcification of the carotid arteries, thoracic aorta, and iliac arteries are predictive of total mortality [24]. Calcification of the aortic valve has a strong association with coronary microvascular endothelial dysfunction and overall coronary atherosclerotic plaque burden as well as MAC [7,8,25]. ACS can be the first manifestation of coronary artery atherosclerosis, thus making the identification of plaques at high risk of complication an important component of strategies to reduce casualties. Approximately 60% of clinically evident plaque ruptures originate from the inflamed TCFA [26]. A previous pathological study demonstrated a relationship between lesion instability and the size of NCs or the presence of TCFAs [27]. Furthermore, the presence of arterial calcium deposits causes compliance mismatch and creates stress failure at the plaque surface, thereby increased the risk of rupture [28].

In this study, the presence of valvular calcification was associated not only with increased calcification, but also with heightened lesion instability in the treated coronary artery segment in the form of VH-TCFA with increased calcium density. Moreover, the combined presence of AVC and MAC and the severity of valvular calcification were significantly associated with vulnerable plaques. Recognizing valvular calcification may help identify individuals with lesion complexity and plaque instability of the coronary arteries, which require aggressive medical intervention such as high-dose statin therapy.

### Study limitations

This study had several limitations. First, this was an observational study with a small sample size. Second, a high proportion of the study population included high risk patients (86% of patients had ACS). Therefore, these results should be applied to the general population with caution. Third, although patients with culprit lesions and prominent thrombi burden were excluded, the presence of a thrombus overlying a culprit lesion could reduce the ability of IVUS to assess underlying plaque characteristics. Thrombectomy itself can affect plaque morphology and the accuracy of the data. Fourth, scant valvular calcification may not be detected due to the low resolution of echocardiography. Fifth, we did not assess the clinical outcome of valvular calcification. Further investigation is necessary to evaluate the clinical impact of valvular calcification. Nonetheless, to the best of our knowledge, the present study is the first to investigate the relationship between valvular calcification and plaque characteristics of the coronary artery assessed using radiofrequency intravascular ultrasound.

## Conclusions

This study found a significant relationship between valvular calcifications and VH-IVUS assessment of TCFA. A high complexity and severity of valvular calcification indicated greater atherosclerotic disease complexity (increased calcification of coronary plaques) as well as vulnerable coronary plaques (higher incidence of VH-TCFA). Further studies are required to evaluate the clinical impact of valvular calcification.

## Supporting Information

S1 Table Patients’ raw data. (XLTX)

S2 Table VH-TCFA (%) in propensity score matching data. (DOCX)

## Abbreviations

ACS: acute coronary syndrome
AVC: aortic valve calcification
CAD: coronary artery disease
DC: dense calcium
FF: fibrofatty
FT: fibrotic tissue
IVUS: intravascular ultrasound
MAC: mitral annular calcification
NC: necrotic core
TCFA: thin-capped fibroatheroma
TTE: transthoracic echocardiography
VH-IVUS: virtual histology intravascular ultrasound
