# Supplementary material for: Plaque Vulnerability as Assessed by Radiofrequency Intravascular Ultrasound in Patients with Valvular Calcification
Source: PLoS One. 2016 Nov 2;11(11):e0165885. doi: 10.1371/journal.pone.0165885 (PMC5091909; doi:10.1371/journal.pone.0165885)
Supplement: S2 Table — (DOCX) [file pone.0165885.s002.docx]

S2 Table. VH-TCFA (%) in propensity score matching data

|  | **No calcification**  **(N=96)** | **Isolated calcification**  **(N=63)** | **Combined presence of**  **AVC and MAC**  **(N=33)** | **p-value** |
| --- | --- | --- | --- | --- |
| VH-TCFA (%) | 38.5 | 31.8 | 60.6 | 0.0064 |
|  | **No calcification**  **(N=96)** | **Mild to moderate**  **calcification**  **(N=48)** | **Severe calcification**  **(N=48)** | **p-value** |
| VH-TCFA (%) | 38.5 | 29.2 | 54.2 | 0.0130 |
